# Epidemiological risk factors for acute kidney injury outcomes in hospitalized adult patients: a multicenter cohort study

**DOI:** 10.1093/ckj/sfae426

**Published:** 2025-01-23

**Authors:** Tomonori Takeuchi, A K M F Rahman, Lama Ghazi, Orson W Moe, Robert D Toto, Edward D Siew, Javier A Neyra, Orlando M Gutierrez

**Affiliations:** Division of Nephrology, Department of Medicine; Heersink School of Medicine, University of Alabama at Birmingham, Birmingham, AL, USA; Department of Health Policy and Informatics; Tokyo Medical and Dental University, Tokyo, Japan; Department of Biostatistics; School of Public Health, University of Alabama at Birmingham, Birmingham, AL, USA; Department of Epidemiology; School of Public Health, University of Alabama at Birmingham, Birmingham, AL, USA; Charles and Jane Pak Center for Mineral Metabolism and Clinical Research, UT Southwestern Medical Center, Dallas, TX, USA; Department of Physiology, UT Southwestern Medical Center, Dallas, TX, USA; Division of Nephrology, Department of Internal Medicine, University of Texas Southwestern Medical Center, Dallas, TX, USA; Charles and Jane Pak Center for Mineral Metabolism and Clinical Research, UT Southwestern Medical Center, Dallas, TX, USA; Division of Nephrology, Department of Internal Medicine, University of Texas Southwestern Medical Center, Dallas, TX, USA; Division of Nephrology and Hypertension, Vanderbilt Center for Kidney Disease, Department of Medicine, Vanderbilt University Medical Center, Nashville, TN, USA; Division of Nephrology, Department of Medicine; Heersink School of Medicine, University of Alabama at Birmingham, Birmingham, AL, USA; Division of Nephrology, Department of Medicine; Heersink School of Medicine, University of Alabama at Birmingham, Birmingham, AL, USA; Department of Epidemiology; School of Public Health, University of Alabama at Birmingham, Birmingham, AL, USA

**Keywords:** acute kidney injury, diabetes mellitus, obesity, race, risk factor

## Abstract

**Background:**

Multiple studies have identified risk factors for acute kidney injury (AKI) in hospitalized patients, but less is known about factors associated with AKI severity, including non-recovery of AKI.

**Methods:**

Retrospective cohort study of adults (≥18 years) hospitalized between 2014 and 2017 at three US academic medical centers. Study outcomes included incidence of AKI and non-recovery from AKI at hospital discharge in those who survived hospitalization. AKI was defined by KDIGO serum creatinine criteria. Non-AKI recovery was defined as persistent AKI stage ≥1 at time of discharge. Multivariable models assessed the association of risk factors for each outcome, focusing on race, diabetes, and obesity (BMI ≥ 30 versus <30 kg/m^2^), and adjusting for potential confounders.

**Results:**

Among 56 056 patients included in the study (mean age 57, 25% Black, 48% women), 12 954 (23%) developed AKI. In adjusted models, Black race [odds ratio (OR) 1.26, 95% confidence interval (CI): 1.20, 1.32], diabetes (OR 1.14, 95% CI: 1.08, 1.19) and obesity (OR 1.14, 95% CI: 1.10, 1.20) were all associated with incident AKI. A total of 3591 of the 11 672 (30.8%) patients with AKI who survived until discharge had AKI non-recovery. In adjusted models, obesity (OR 1.27, 95% CI: 1.17, 1.39) was independently associated with higher risk of AKI non-recovery at hospital discharge.

**Conclusions:**

Black race, diabetes, and obesity were associated with the development of AKI in hospitalized patients, but only obesity was associated with non-recovery from AKI at hospital discharge. These findings emphasize the growing relevance of obesity as an epidemiological risk factor of AKI.

KEY LEARNING POINTS **What was known:**

- Previous studies primarily focused on the relationship of epidemiological factors such as obesity, diabetes, and race with incident AKI.
- The relationships of these factors with non-recovery from AKI at discharge were seldom examined.

**This study adds:**

- This study identifies obesity as a risk factor not only for development of AKI during hospitalization but also for non-recovery from AKI at discharge.
- Black race and diabetes were associated with overall incident AKI but not with non-recovery from AKI.

**Potential impact:**

- These findings can improve risk stratification for hospitalized patients with AKI.
- Enhanced post-discharge follow-up for obese patients who survived an episode of AKI may attenuate the burden of chronic kidney disease after AKI.

## INTRODUCTION

Acute kidney injury (AKI) is common in hospitalized patients and is associated with excess morbidity and mortality [1]. Although most prior studies examining the epidemiology of AKI in the US have focused on risk factors for the development of any stage of AKI in hospitalized patients [2, 3], few studies have examined factors associated with the severity of AKI or adverse AKI outcomes, such as non-recovery of AKI in patients who survive up to discharge. A better understanding of these factors may be helpful to develop strategies for preventing, managing, and following high-risk AKI patients, as well as mitigating the risk of chronic kidney disease (CKD) after AKI.

In this study, we aimed to identify factors associated with the severity of AKI and non-recovery of AKI in patients hospitalized at three large academic medical centers in the US. We focused particularly on Black race, diabetes, and obesity because these factors are most strongly associated with the prevalence of CKD in the Southeastern US, and are thought to play a large role in explaining excess rates of adverse health outcomes in this region as compared to other regions of the country [4–6]. We hypothesized that these risk factors would be independently associated with the severity of AKI during hospitalization and would associate with AKI non-recovery in those who survive to hospital discharge, which could aid future efforts to identify factors for AKI risk-classification specific to areas of high prevalence of AKI and CKD.

## MATERIALS AND METHODS

### Study design and population

We conducted a multicenter retrospective cohort study at the University of Alabama at Birmingham (UAB, Birmingham, Alabama), the University of Kentucky (Lexington, Kentucky), and the University of Texas Southwestern (Dallas, Texas). Electronic health record (EHR) data were extracted from the data

warehouse of each medical center using a common data dictionary. We included individuals admitted to one of the three hospitals from 1 October 2014 to 30 September 2017 with at least four serum creatinine measurements during the index hospitalization, for the purpose of calculating baseline kidney function and outcomes. We excluded individuals who were less than 18 years old, prisoners, patients transferred from other hospitals, patients who were admitted for same-day procedures, and individuals with end-stage kidney disease (ESKD), including those on maintenance dialysis or kidney transplant (identified through ICD-9 or -10 codes; Supplementary Table S1). This study was approved by the Institutional Review Board of each institution and waivers of informed consent were obtained given the retrospective nature of the investigation.

### Independent variables

The primary independent variables of interest were Black race (versus other), diabetes status (yes or no), and obesity status (yes or no) as these factors are among the strongest risk factors for CKD in the Southeastern US. Race was defined using variables contained in the EHR. Diabetes was defined as having either type 1 or type 2 diabetes using ICD-9 and ICD-10 codes (Supplementary Table S1), whereas obesity was defined as a body mass index (BMI) ≥ 30 kg/m² using data from the index hospitalization.

### Study outcomes

The primary outcome was the development and severity of AKI during the hospitalization using serum creatinine-based KDIGO criteria [7]. All serum creatinine measurements were extracted from the EHR. AKI stage 1 was defined as an increase in serum creatinine ≥0.3 mg/dl or ≥150–199% of baseline; AKI stage 2 as an increase in serum creatinine to 200–299% of baseline; and AKI stage 3 as an increase of serum creatinine ≥300% of baseline or initiation of dialysis. Because we did not have outpatient serum creatinine values prior to the index hospitalization in all three sites reliably, baseline serum creatinine was defined as the lowest value of the first three inpatient values measured while not on dialysis, as has been done before [1, 8], with all subsequent serum creatinine values beyond the first three being used to identify AKI. A secondary outcome was non-recovery of AKI by the time of hospital discharge, defined as the last serum creatinine being >25% or ≥0.3 mg/dl of the baseline value in patients with AKI not on dialysis at the time of evaluation or those receiving dialysis within the last 72 hours prior to discharge.

### Covariates

Data related to demographics (age, sex) and comorbidities were extracted from the EHR. Specific comorbidities were determined based on ICD-9 and ICD-10 codes to calculate the Elixhauser comorbidity score [9]. Baseline estimated glomerular filtration rate (eGFR) was calculated using the 2009 CKD-EPI creatinine equation, using the baseline serum creatinine [10].

### Statistical analyses

To summarize the baseline patient characteristics, including demographics and comorbidities, of those who developed AKI compared to those who did not, we used *t*-tests to compare the means of continuous variables and χ^2^ tests to compare the proportions of categorical variables. We also used the Wilcoxon rank-sum test to compare the medians between groups for non-normally distributed measures. Binomial logistic regression models were used to examine the associations of Black versus other race, diabetes (yes versus no), and obesity (yes versus no) with incident AKI; and multinomial logistic regression models were used to examine the associations of Black race, diabetes, and obesity with AKI severity (KDIGO stages 1–3). To account for potential confounding factors, we examined sequentially adjusted models. Model 1 adjusted for site only. Model 2 further adjusted for age, sex, baseline eGFR, and Black race (when diabetes and obesity were the main exposure variable), diabetes (when Black race and obesity were the main exposure variables), and obesity (when Black race and diabetes were the main exposure variables). Model 3 adjusted for all variables in Model 2 plus the Elixhauser comorbidity score. Logistic regression models were used to examine the association Black race, obesity and diabetes with AKI non-recovery in those who survived to hospital discharge using the exact same modeling as outlined above.

### Sensitivity analyses

With data from one study site (UAB), a sensitivity analysis was conducted by defining baseline serum creatinine (SCr) as the most recent outpatient SCr measured within 7 days to 1 year prior to the index admission. If no outpatient SCr was available, the most recent inpatient SCr measured within the same time frame was used. The sensitivity analysis replicated the logistic regression models described above for AKI incidence and AKI stages. In addition to Models 2 and 3, we examined Model 4, which was adjusted for all variables in Model 3, as well as pre-admission use of loop diuretics, use of angiotensin-converting enzyme inhibitors (ACE-i) or angiotensin II receptor blockers (ARB), pre-admission use of mineralocorticoid receptor antagonists (MRA), the average of mean arterial pressure during the first 48 hours of hospitalization, exposure to nephrotoxins during the first 48 hours of hospitalization, exposure to intravascular contrast agents during the first 48 hours of hospitalization, and admission to a surgical intensive care unit (ICU). Nephrotoxins included aminoglycosides, vancomycin, non-steroidal anti-inflammatory drugs (NSAIDs), calcineurin inhibitors, and amphotericin B. Additionally, as detailed SCr timestamps were available for this subcohort, we performed a multivariable Cox proportional hazards model analysis with time to AKI recovery as the outcome among survivors who developed AKI. The time window for loop diuretics, ACE-i, ARB, and MRA exposure was adjusted to include only exposures occurring prior to AKI onset during hospitalization. Additional covariates mirrored those of prior models.

All tests were two-sided and a *P*-value of <.05 was considered statistically significant. The main analyses were performed using SAS version 9.4 software (SAS Institute, Inc.; Cary, NC) and sensitivity analyses were conducted with R version 4.2.2 (The R Foundation for Statistical Computing, Vienna, Austria). REGARDS data are not publicly available due to ethical and legal restrictions. To abide by its obligations with NIH/NINDS and the Institutional Review Board of the University of Alabama at Birmingham, REGARDS facilitates data sharing through data use agreements. Any investigator is welcome to access the REGARDS data, including statistical code, through this process. Requests for data access may be sent to regardsadmin@uab.edu.

## RESULTS

### Study population characteristics

**Figure 1:**
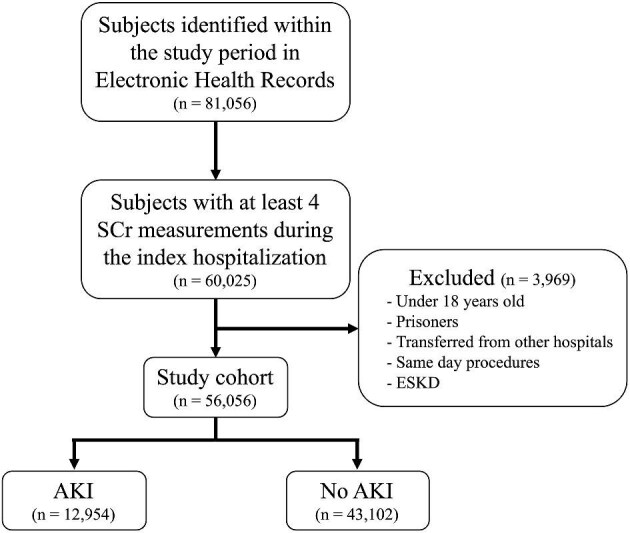
Flow diagram of patients represented in this study.

Of the 56 056 adult patients in the study cohort, a total of 12 954 (23%) developed AKI (Fig. 1). The mean age of patients who developed AKI was 59 (SD 17) years, 44% were women and 29% were Black. The prevalence of diabetes and obesity in patients with AKI were 27% and 33%, respectively. Among patients without AKI (*n* = 43 102), the mean age was 57 (SD 18) years, 50% were women, and 24% were Black. The prevalence of diabetes and obesity in patients without AKI were 20% and 27%, respectively (Table 1).

**Figure 2:**
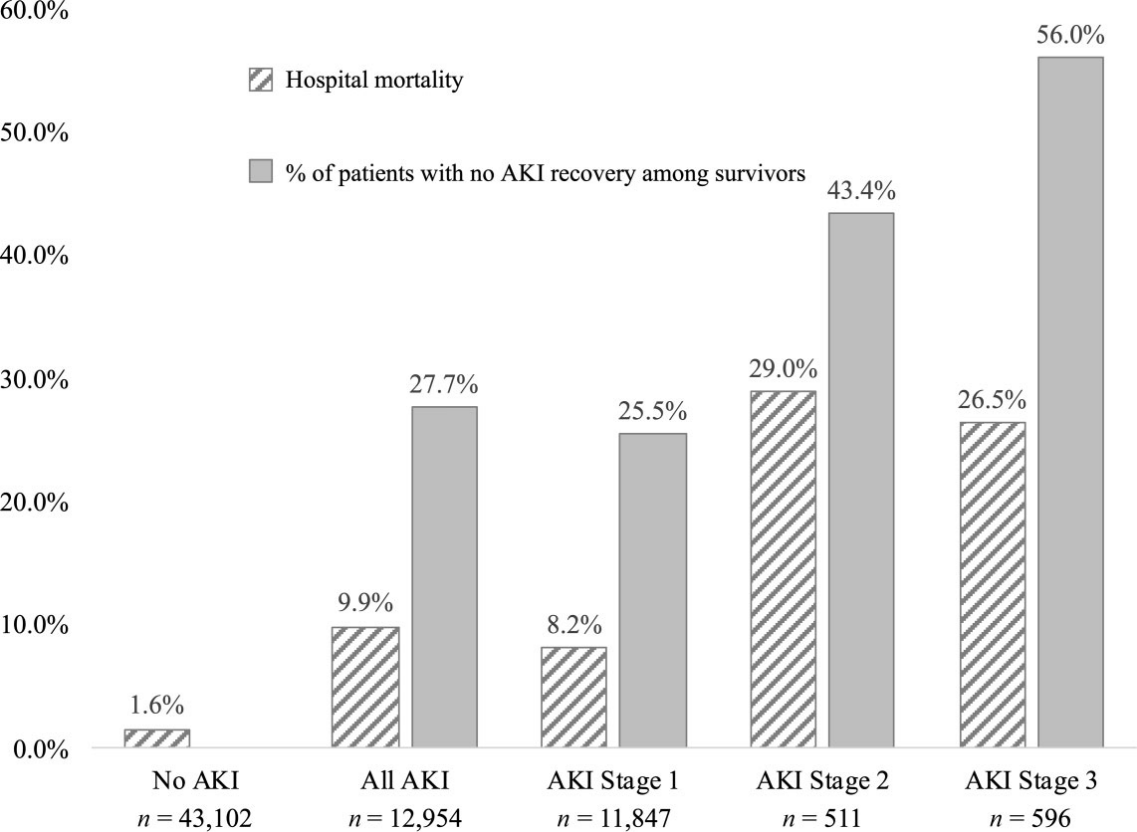
Hospital mortality and no acute kidney injury (AKI) recovery by AKI severity stage.

**Table 1:** Characteristics of study participants by acute kidney injury status.

Characteristics	AKI	No AKI
*N* (%) *N* = 56 056	12 954 (23.1)	43 102 (76.9)
Age, mean (SD)	59.0 (17.0)	56.71 (17.8)
Male sex, *N* (%)	7268 (56.1)	21 652 (50.2)
Black race, *N* (%)	3694 (28.5)	10 142 (23.5)
Diabetes, *N* (%)	3437 (26.5)	8687 (20.2)
BMI, kg/m^2^, mean (SD)	30.0 (8.4)	29.1 (8.1)
BMI, kg/m^2^, median (IQR)	28.4 (24.3, 33.9)	27.6 (23.6, 32.9)
Obesity (BMI ≥ 30 kg/m^2^), *N* (%)	4222 (32.6)	11 752 (27.2)
Serum creatinine, mg/dl, median (IQR)	1.0 (0.7, 1.4)	0.8 (0.6, 1.0)
eGFR, ml/min/1.73 m^2^, median (IQR)	76.2 (47.7, 101.9)	92.3 (68.8, 110.5)
Systolic blood pressure, mean (SD)	133.0 (30.5)	133.0 (25.8)
Diastolic blood pressure, mean (SD)	75.8 (20.7)	76.2 (17.0)
Length of hospital stay, median (IQR)	8.0 (4.9, 14.2)	5.0 (3.0, 8.0)
Elixhauser Comorbidity Score, mean (SD)	16.7 (13.7)	10.2 (11.7)
Elixhauser comorbidities, *N* (%)		
Hypertension without complications	5853 (45.2)	19 508 (45.3)
Hypertension with complications	3268 (25.2)	5053 (11.7)
Congestive heart failure	3457 (26.7)	6005 (13.9)
Cardiac arrhythmia	2840 (21.9)	6868 (15.9)
Valvular heart disease	1440 (11.12)	2823 (6.6)
Pulmonary circulation disease	1265 (9.8)	2119 (4.9)
Peripheral vascular disease	1385 (10.7)	3136 (7.3)
Paralysis	284 (2.2)	936 (2.2)
Other neurologic disorder	1336 (10.3)	3821 (8.9)
Chronic pulmonary disease	2893 (22.3)	8357 (19.4)
Hypothyroidism	1598 (12.3)	4771 (11.1)
Chronic kidney disease	3204 (24.7)	4349 (10.1)
Liver disease	1574 (12.2)	3379 (7.8)
Peptic ulcer disease	68 (0.5)	279 (0.7)
Acute immunodeficiency syndrome	146 (1.1)	393 (0.9)
Lymphoma	241 (1.86)	931 (2.2)
Metastatic cancer	902 (7.0)	3209 (7.5)
Solid tumor without metastasis	1800 (13.9)	5884 (13.7)
Rheumatoid arthritis	459 (3.5)	1518 (3.5)
Coagulopathy	1693 (13.1)	2746 (6.4)
Weight loss	2284 (17.7)	4193 (9.7)
Fluid and electrolyte disorder	8367 (64.6)	19 350 (44.9)
Blood loss anemia	186 (1.4)	513 (1.2)
Deficiency anemias	533 (4.1)	1331 (3.1)
Alcohol abuse	779 (6.0)	2458 (5.7)
Drug abuse	727 (5.6)	2622 (6.1)
Psychoses	215 (1.7)	732 (1.7)
Depression	2121 (16.4)	6703 (15.6)

BMI, body mass index; eGFR, estimated glomerular filtration rate; IQR, interquartile range.

In general, as compared to patients without AKI, patients with AKI were older, were more frequently men and Black, had a higher prevalence of diabetes and obesity, had a lower baseline eGFR, and had a greater burden of comorbidities at time of hospital admission, as evidenced by a higher Elixhauser scores (Table 1). A total of 11 847 patients (92%) had AKI stage 1, 511 (4%) had AKI stage 2, and 596 (5%) had AKI stage 3 (Fig. 2). A total of 486 (3.8%) patients required any form of dialysis for AKI support during the hospitalization.

Patients with AKI had higher hospital mortality than those without AKI (10% versus 2%, *P* < .001). The mortality rates were higher in those with more severe AKI: 8%, 29%, and 27% for AKI stages 1, 2 and 3, respectively. Among 13 058 patients from the UAB subcohort with preadmission SCr data, 3893 (29.8%) developed AKI. The hospital mortality rates and the proportion of patients with no recovery from AKI according to AKI stages are reported in Supplementary Fig. S1.

### Factors associated with incidence and severity of AKI in hospitalized patients

**Figure 3:**
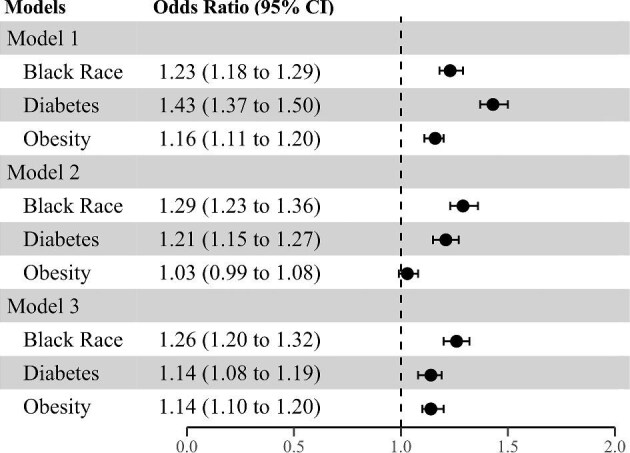
Association of Black race, diabetes, and obesity with development of acute kidney injury (AKI) in hospitalized patients. Model 1 is adjusted for study site. Model 2 is adjusted for age, sex, study site, Black race (when diabetes and obesity were main exposure variables), diabetes (when race and obesity were the main exposure variables), obesity (when race and diabetes were the main exposure variables), and baseline estimated glomerular filtration rate. Model 3 is adjusted for all variables in Model 2 plus the Elixhauser comorbidity score.

In the model adjusted for study site alone (Model 1), Black race [odds ratio (OR) 1.23, 95% confidence interval (CI) 1.18, 1.29], diabetes (OR 1.43, 95% CI 1.37, 1.50), and obesity (OR 1.16, 95% CI 1.11, 1.20) were associated with higher odds of incident AKI (Fig. 3). The magnitude and strength of the associations did not substantively differ after further adjustment for age, sex, baseline eGFR, race, diabetes, and obesity as appropriate depending on the model (Model 2), or in the adjusted model including the Elixhauser comorbidity score (Model 3). In the multivariable model (Model 3), in addition to Black race, diabetes and obesity, youngerage, higher Elixhauser comorbidity score, lower baseline eGFR, and male sex were all independently associated with higher occurrence of AKI (Supplementary Table S2a).

**Figure 4:**
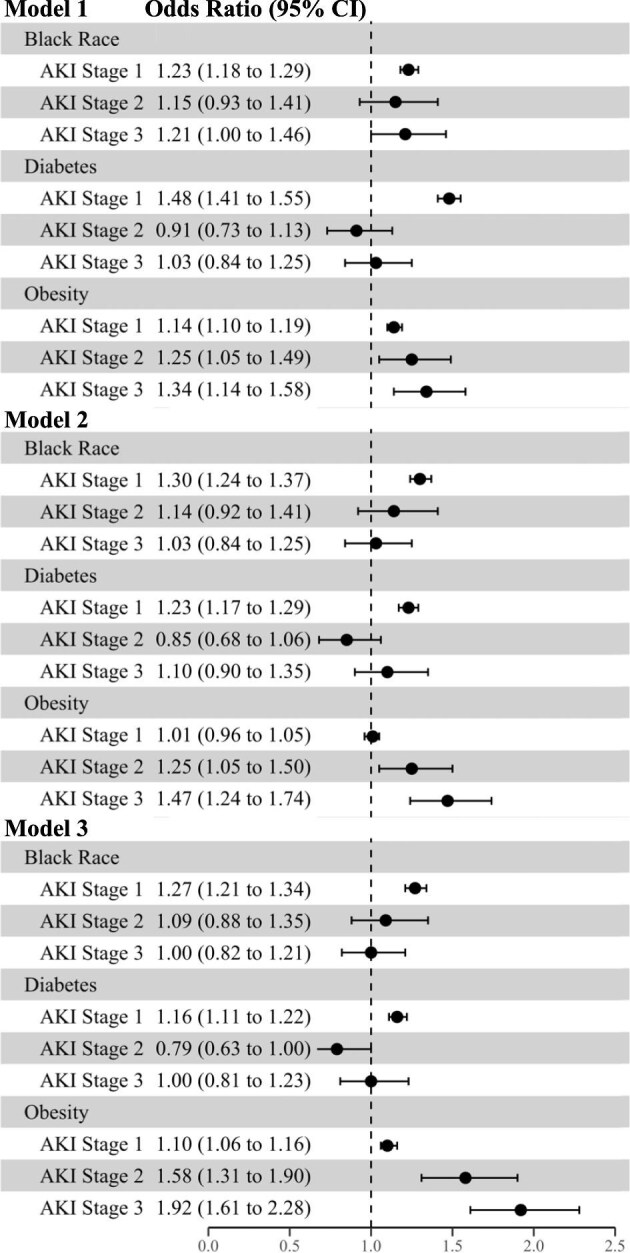
Association of Black race, diabetes, and obesity with development of acute kidney injury stage 1, 2, or 3 versus no acute kidney injury in hospitalized patients. Model 1 is adjusted for study site. Model 2 is adjusted for age, sex, study site, Black race (when diabetes and obesity were main exposure variables), diabetes (when race and obesity were the main exposure variables), obesity (when race and diabetes were the main exposure variables), and baseline estimated glomerular filtration rate. Model 3 is adjusted for all variables in Model 2 plus the Elixhauser comorbidity score.

In multinomial models examining severity of AKI (stage 1, 2, or 3 versus no AKI) as the outcome variable, Black race and diabetes were associated with higher odds of developing stage 1 AKI in all models (Fig. 4), but there were no statistically significant associations of Black race or diabetes with the development of stage 2 or 3 AKI in adjusted models. In contrast, obesity was independently associated with higher odds of developing AKI stage 1, 2, or 3 in all models, such that in the adjusted model, patients with obesity had nearly twofold higher odds of developing stage 3 AKI as compared to patients without obesity (Fig. 4). In secondary analyses stratified by site, the results were similar by site (Supplementary Table S3). In the final multivariable model, similar to obesity, higher Elixhauser comorbidity score was independently associated with greater odds of AKI stages 1, 2, and 3 (Supplementary Table S2b).

Sensitivity analyses with additional model covariates (Model 4) confirmed the association of Black race, diabetes, and obesity with the development of AKI during hospitalization (Supplementary Table S4), but also showed a significant association of Black race, diabetes and obesity with severe AKI (stages 2 or 3) (Supplementary Table S5).

### Factors associated with non-recovery of AKI in hospitalized patients

**Figure 5:**
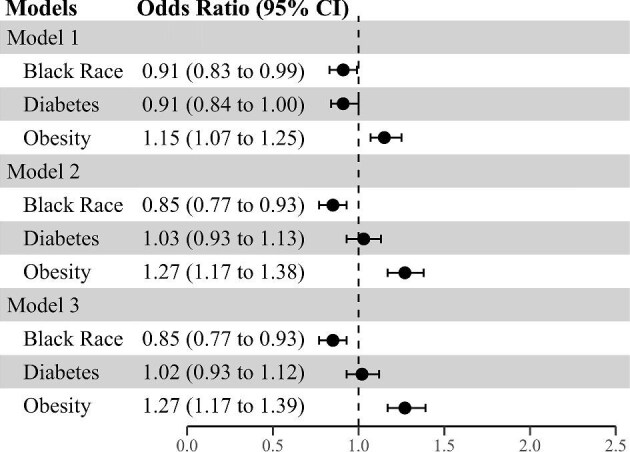
Association of Black race, diabetes, and obesity with no acute kidney injury (AKI) recovery in hospitalized patients who survived to discharge. Model 1 adjusted for study site. Model 2 is adjusted for age, sex, study site, Black race (when diabetes and obesity were main exposure variables), diabetes (when race and obesity were the main exposure variables), obesity (when race and diabetes were the main exposure variables), and baseline estimated glomerular filtration rate. Model 3 is adjusted for all variables in Model 2 plus the Elixhauser comorbidity score.

Of the 12 954 hospitalized patients with AKI, 11 672 survived up to discharge. Of these, a total of 3591 (31%) did not recover kidney function by the time of hospital discharge. The percentage of individuals who did not recover from AKI increased in a step-wise manner with each higher stage of AKI severity (Fig. 2). Black race was independently associated with lower odds of non-recovery of AKI by hospital discharge in all models (Fig. 5), such that in Model 3, Black race was associated with 15% lower odds of non-recovery of AKI (OR 0.85, 95% CI 0.77, 0.93). In contrast, obesity was associated with higher odds of non-recovery of AKI in adjusted models (OR 1.27, 95% CI 1.17, 1.39 in Model 3). There was no association of diabetes with non-recovery of AKI in any model (Fig. 5). In Model 3, other factors associated with non-recovery of AKI were older age, higher baseline eGFR, and female sex (Supplementary Table S2c).

Sensitivity analyses using time-to-recovery in the UAB subcohort with measured pre-admission baseline SCr confirmed the significant association of obesity with lower chances of AKI recovery at discharge (Supplementary Table S6). However, Black race was not associated with higher chances of AKI recovery in the adjusted models (hazard ratio 0.97, 95% CI 0.89, 1.06; Supplementary Table S6).

## DISCUSSION

In this analysis of hospitalized patients at three large academic health centers in the US, we found that Black race, diabetes, and obesity were associated with incident AKI in hospitalized patients. Further, obesity was also independently associated with non-recovery of AKI in those who survived to hospital discharge. These findings suggest that obesity is a key risk factor for poor AKI outcomes, which may help inform efforts for identifying individuals who might benefit the most from post-discharge AKI follow-up.

Prior studies have shown that obesity is associated with incident AKI. In an observational study using MIMIC-III, a significant association between obesity and AKI in critically ill patients was demonstrated. Furthermore, similar to our study, obesity was associated with AKI stages 2 and 3 based on KDIGO serum creatinine criteria, and the association was stronger with higher BMI [11]. In another single-center retrospective study using RIFLE criteria to define AKI, obesity was also found to be associated with AKI [12]. Our findings add to these prior studies in two ways. First, we examined all hospitalized patients from three large academic medical centers and did not focus solely on critically ill populations or those who were undergoing cardiac or bariatric surgery as was the case in prior studies. Thus, our findings indicate that the association of obesity with the incidence and severity of AKI is generalizable to hospitalized patients beyond those who are critically ill or undergoing specific surgeries. Second, our study evaluated the association between obesity and AKI recovery and showed that obesity was also independently associated with non-recovery of AKI in those who survived to discharge. This finding may aid in creating risk-stratification models for identifying individuals least likely to recover from AKI and for whom post-hospitalization follow-up may be most beneficial and cost-effective.

The observed association between obesity and AKI can be explained by the potential burden of comorbidity influenced by obesity. In a study involving cardiac surgery patients (*n* = 445), a regression model revealed an association between AKI and obesity, which disappeared after adjusting for the factor of oxidative stress, suggesting a significant involvement of oxidative stress in the etiology of AKI in obese patients [13]. However, obesity is a complex multifactorial condition with various underlying causes and effects, which must be taken into consideration when discussing its potential impact on the development and outcomes of AKI. Socioeconomic factors have been shown to have an inverse correlation with obesity and are also associated with decreased kidney function [14–18]. Assuming that obesity serves as a proxy variable reflecting social determinants of health, the results of this study do not necessarily negate the possibility that social determinants of health may be additional key risk factors for adverse AKI outcomes. Further, although observational studies have suggested that acutely ill obese (versus normal weight) patients may have a lower risk of hospital mortality [19], the mechanisms of this ‘obesity paradox’ and how AKI could disentangle this relationship (hospital mortality rates in AKI patients with BMI ≥30 versus <30 in our cohort: 9.2% versus10.4%, *P*-value = .03) are still not fully understood.

Prior studies have shown that Black individuals are at higher risk of developing AKI than non-Black populations [20, 21]. Our study adds to this literature by showing that whereas Black individuals are at higher risk of hospitalized AKI, they do not appear to have higher risk of non-recovery from AKI by discharge. Prevalent diabetes has long been identified as a risk factor for AKI, potentially involving the influence of complications linked to diabetes and susceptibility to treatment and dehydration [22–25]. However, whether diabetes is associated with recovery of AKI has not been thoroughly investigated. In this study, diabetes was associated with higher risk of hospitalized AKI but not with recovery of AKI. As this study relied on ICD codes for the determination of diabetes, there is a possibility that the prevalence of diabetes was underestimated, and the results may differ depending on the type and stage of diabetes.

This study has several limitations. First, being an observational study, unmeasured confounding factors may exist. However, we attempted to adjust our models for clinically and epidemiologically important covariates. There may be variations in socioeconomic status not only between regions but also between households in the areas we studied, which could have influenced the results. However, evaluation of social determinants of health was beyond the scope of this manuscript and subject of future work by our group. The main study cohort did not have pre-admission creatinine measurements to use as baseline, which was determined by examination of inpatient values. However, sensitivity analyses using a subcohort with pre-admission values showed similar findings as the main analyses. Our study also had several key strengths, including utilizing multicenter and harmonized data from three large academic centers. Further, we did utilize contemporary definitions of exposures and outcomes. Finally, the observation that the analytic results were replicated in all three centers attests to the robustness of the results and conclusions.

To conclude, in this large cohort study of hospitalized patients in three academic centers, we demonstrated that Black race, diabetes, and obesity were associated with incident hospitalized AKI, but only obesity was associated with non-recovery of AKI at hospital discharge. Importantly, these data provide insight into obesity as a risk factor that may be driving adverse AKI outcomes, with potential for improving risk-classification of obese individuals discharged from the hospital following an AKI episode.

## Supplementary Material

sfae426_Supplemental_File

## Data Availability

Data utilized and reported in this manuscript will be available upon request.
