# Supplementary material for: Epidemiological risk factors for acute kidney injury outcomes in hospitalized adult patients: a multicenter cohort study
Source: Clin Kidney J. 2025 Jan 23;18(2):sfae426. doi: 10.1093/ckj/sfae426 (PMC11811520; doi:10.1093/ckj/sfae426)
Supplement: sfae426_Supplemental_File [file sfae426_supplemental_file.pdf]

Supplementary Material

Table of contents

1. **Table S1. ICD-9/ ICD-10 Codes for Kidney Disease and Elixhauser Comorbidity**
2. **Figure S1. Hospital mortality and no AKI recovery by AKI severity stage in the sub-cohort from the University of Alabama at Birmingham for sensitivity analyses**
3. **Table S2. Outputs of the fully adjustment models for each outcome**
   a. **Association of each factor with development of AKI in hospitalized patients**
   b. **Association of each factor with development of acute kidney injury stage 1, 2, or 3 vs. no acute kidney injury in hospitalized patients**
   c. **Association of each factor with no AKI recovery in hospitalized patients who survived to discharge**
4. **Table S3. Association of Black race, diabetes and obesity with development of acute kidney injury stratified by stage in hospitalized patients.**
   a. **The University of Alabama at Birmingham**
   b. **The University of Kentucky Medical Center**
   c. **The University of Texas Southwestern**
5. **Table S4: Association of Black race, diabetes, and obesity with development of AKI in hospitalized patients using pre-admission serum creatinine for baseline definition (cohort from the University of Alabama at Birmingham)**
6. **Table S5: Association of Black race, diabetes, and obesity with development of AKI stratified by stage in hospitalized patients using pre-admission serum creatinine for baseline definition (cohort from the University of Alabama at Birmingham)**
7. **Table S6: Association of Black race, diabetes, and obesity with AKI recovery in hospitalized patients who survived to discharge using pre-admission serum creatinine for baseline definition (cohort from the University of Alabama at Birmingham)**
8. **STROBE Statement - Checklist**

Table S1. ICD-9/ ICD-10 Codes for Kidney Disease and Elixhauser Comorbidity Score

**Kidney Disease**	**ICD-9/ 1CD-10**	**Codes**
**End Stage Kidney Disease**	ICD-9	585.6'
	ICD-10	N18.6'
**Kidney Transplant**	ICD-9	996.81'
	ICD-10	Z94.0', 'V42.0', 'Z48.22', 'T86.12', 'T86.11', 'T86.10'
**Elixhauser Comorbidity**	**ICD-9/ 1CD-10**	**Codes**
**Congestive Heart Failure**	ICD-9	39891','40201','40211','40291','40401','40403','40411','40413','40491', '40493','4254','4255','4257','4258','4259','428'
	ICD-10	I099','I110','I130','I132','I255','I420','I425','I426','I427','I428', 'I429','I43','I50','P290'
**Cardiac Arrhythmia**	ICD-9	4260','42613','4267','4269','42610','42612','4270','4271','4272','427 3','4274','4276','4278','4279','7850','99601','99604','V450','V533'
	ICD-10	I441','I442','I443','I456','I459','I47','I48','I49','R000','R001','R008','T821','Z 450','Z950'
**Valvular Heart Disease**	ICD-9	0932','394','395','396','397','424','7463','7464','7465','7466','V422','V433'
	ICD-10	A520','I05','I06','I07','I08','I091','I098','I34','I35','I36','I37','I38','I39','Q230', 'Q231','Q232','Q233','Z952','Z953','Z954'
**Pulmonary Circulation Disorder**	ICD-9	4150','4151','416','4170','4178','4179'
	ICD-10	I26','I27','I280','I288','I289'
**Peripheral Vascular Disease**	ICD-9	0930','4373','440','441','4431','4432','4438','4439','4471','5571','5579','V43 4'
	ICD-10	I70','I71','I731','I738','I739','I771','I790','I792','K551','K558', 'K559','Z958','Z959'
**Hypertension**	ICD-9	401', '402','403','404','405'
	ICD-10	I10', 'I11','I12','I13','I15'
**Paralysis**	ICD-9	3341','342','343','3440','3441','3442','3443','3444','3445','3446','3449'
	ICD-10	G041','G114','G801','G802','G81','G82','G830','G831','G832','G833', 'G834','G839'
**Other Neurological Disorder**	ICD-9	3319','3320','3321','3334','3335','33392','334','335','3362','340','341','345','3 481','3483','7803','7843'
	ICD-10	G10','G11','G12','G13','G20','G21','G22','G254','G255','G312','G318','G31 9','G32','G35','G36','G37','G40','G41','G931','G934','R470','R56'

**Chronic Pulmonary Disease**	ICD-9	4168','4169','490','491','492','493','494','495','496','500','501','502', '503','504','505','5064','5081','5088
	ICD-10	I278','I279','J40','J41','J42','J43','J44','J45','J46','J47','J60','J61', 'J62','J63','J64','J65','J66','J67','J684','J701','J703'
**Diabetes, Uncomplicated**	ICD-9	250.0','250.1','250.2','250.3'
	ICD-10	E10.0','E10.1','E10.9','E11.0','E11.1','E11.9','E12.0','E12.1','E12.9','E13.0', 'E13.1','E13.9','E14.0','E14.1','E14.9'
**Diabetes, Complicated**	ICD-9	250.4','250.5','250.6','250.7','250.8','250.9'
	ICD-10	E10.2','E10.3','E10.4','E10.5','E10.6','E10.7','E10.8','E11.2','E11.3','E11.4',' E11.5','E11.6','E11.7','E11.8','E12.2','E12.3','E12.4','E12.5','E12.6','E12.7',' E12.8','E13.2','E13.3','E13.4','E13.5','E13.6','E13.7','E13.8','E14.2','E14.3',' E14.4','E14.5','E14.6', 'E14.7','E14.8'
**Hypothyroidism**	ICD-9	2409','243','244','2461','2468'
	ICD-10	E00','E01','E02','E03','E890'
**Renal Failure (**Chronic kidney disease)	ICD-9	40301','40311','40391','40402','40403','40412','40413','40492','40493','585' ,'586','5880','V420','V451','V56'
	ICD-10	I120','I131','N18','N19','N250','Z490','Z491','Z492','Z940','Z992'
**Liver Disease**	ICD-9	07022','07023','07032','07033','07044','07054','0706','0709','4560','4561','4 562','570','571','5722','5723','5724','5728','5733','5734','5738','5739','V427'
	ICD-10	B18','I85','I864','I982','K70','K711','K713','K714','K715','K717','K72','K73' ,'K74','K760','K762','K763','K764','K765','K766','K767','K768','K769','Z9 44'
**Peptic Ulcer Disease**	ICD-9	5317','5319','5327','5329','5337','5339','5347','5349'
	ICD-10	K257','K259','K267','K269','K277','K279','K287','K289'
**AIDS/HIV**	ICD-9	042','043','044'
	ICD-10	B20','B21','B22','B24'
**Lymphoma**	ICD-9	200','201','202','2030','2386'
	ICD-10	C81','C82','C83','C84','C85','C88','C96','C900','C902'
**Metastatic Cancer**	ICD-9	196','197','198','199'
	ICD-10	C77','C78','C79','C80'
**Solid Tumor without Metastasis**	ICD-9	140','141','142','143','144','145','146','147','148','149','150','151','152', '153','154','155','156','157','158','159','160','161','162','163','164','165','166',' 167','168','169','170','171','172','174','175','176','177','178','179','180','181',' 182','183','184','185','186','187','188','189','190','191','192','193','194','195'
	ICD-10	C00','C01','C02','C03','C04','C05','C06','C07','C08','C09','C10','C11','C12',' C13','C14','C15','C16','C17','C18','C19','C20','C21','C22','C23','C24','C25','

		C26','C30','C31','C32','C33','C34','C37','C38','C39','C40','C41','C43','C45',' C46','C47','C48','C49','C50','C51','C52','C53','C54','C55','C56','C57','C58',' C60','C61','C62','C63','C64','C65','C66','C67','C68','C69','C70','C71','C72',' C73','C74','C75','C76','C97'
**Rheumatoid Arthritis**	ICD-9	446','7010','7100','7101','7102','7103','7104','7108','7109','7112','714', '7193','720','725','7285','72889','72930'
	ICD-10	L940','L941','L943','M05','M06','M08','M120','M123','M30','M310','M311' ,'M312','M313', 'M32','M33','M34','M35','M45','M461','M468','M469'
**Coagulopathy**	ICD-9	286','2871','2873','2874','2875'
	ICD-10	D65','D66','D67','D68','D691','D693','D694','D695','D696'
**Obesity**	ICD-9	278.0
	ICD-10	E66'
**Weight Loss**	ICD-9	260','261','262','263','7832','7994'
	ICD-10	E40','E41','E42','E43','E44','E45','E46','R634','R64'
**Fluid, Electrolyte, and Nutrition**	ICD-9	2536','276'
	ICD-10	E222','E86','E87'
**Blood Loss Anemia**	ICD-9	280.0'
	ICD-10	D50.0'
**Deficiency Anemia**	ICD-9	2801','2808','2809','281'
	ICD-10	D508','D509','D51','D52','D53'
**Alcohol Abuse**	ICD-9	2652','2911','2912','2913','2915','2918','2919','3030','3039','3050', '3575','4255','5353','5710','5711','5712','5713','980','V113'
	ICD-10	F10','E52','G621','I426','K292','K700','K703','K709','T51','Z502','Z714','Z721'
**Drug Abuse**	ICD-9	292','304','3052','3053','3054','3055','3056','3057','3058','3059','V6542'
	ICD-10	F11','F12','F13','F14','F15','F16','F18','F19','Z715','Z722'
**Psychoses**	ICD-9	2938','295','29604','29614','29644','29654','297','298'
	ICD-10	F20','F22','F23','F24','F25','F28','F29','F302','F312','F315'
**Depression**	ICD-9	2962','2963','2965','3004','309','311'
	ICD-10	F204','F313','F314','F315','F32','F33','F341','F412','F432'

Figure S1. Hospital mortality and no AKI recovery by AKI severity stage in the sub-cohort from the University of Alabama at Birmingham for sensitivity analyses

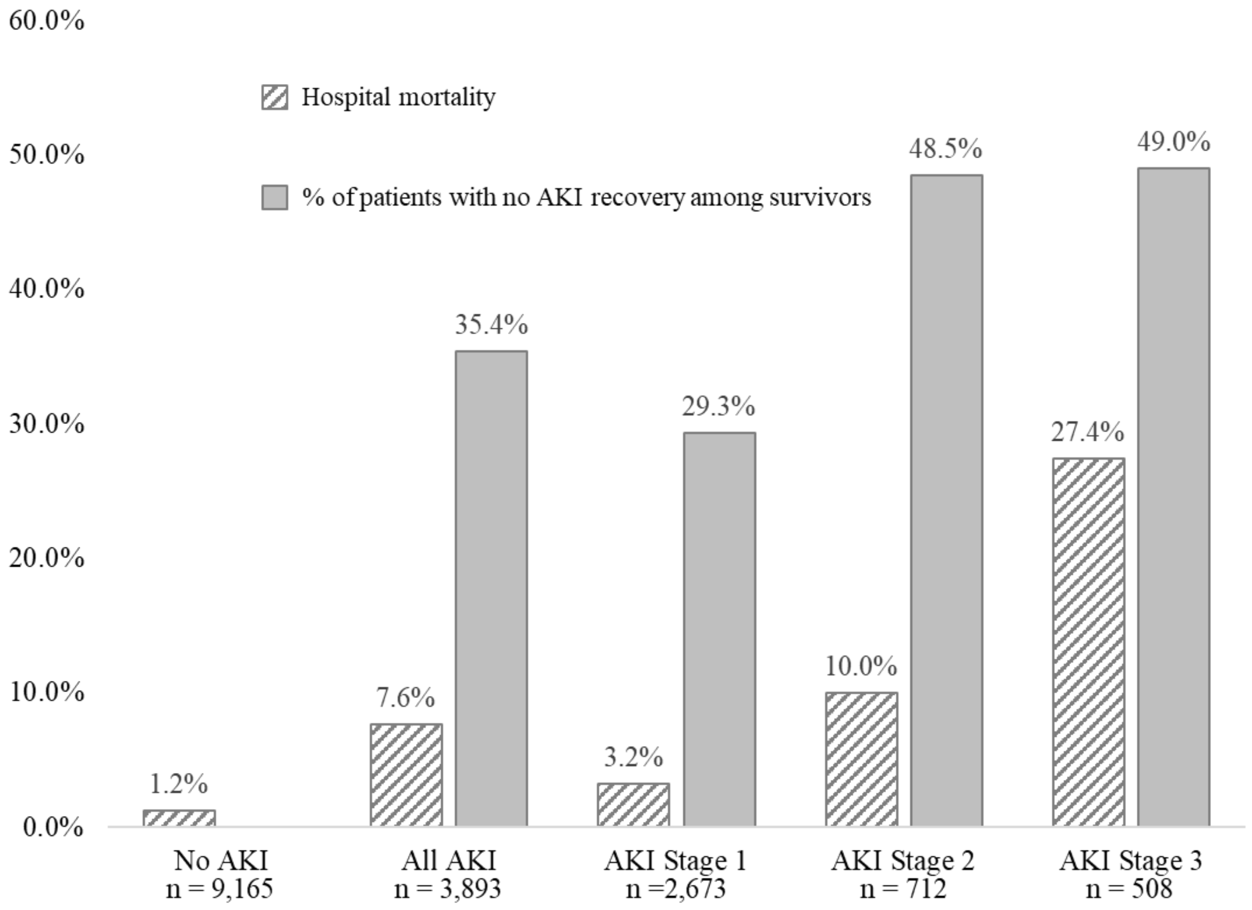

Abbreviations: AKI, acute kidney injury

Table S2: Outputs of the fully adjusted models for each study outcome

Table S2 – a: Association of each factor with development of AKI in hospitalized patients

Independent variables	OR	(95% CI)
Black race (vs. other)	1.260	(1.200 - 1.322)
Diabetes (yes vs. no)	1.137	(1.082 - 1.194)
Obesity (BMI ≥30 vs. <30 kg/m2)	1.144	(1.095 - 1.195)
Age (per 1-year older)	0.990	(0.989 - 0.992)
Sex (male vs. female)	1.179	(1.131 - 1.229)
Baseline eGFR (per 1-unit increase)	0.988	(0.987 - 0.989)
Elixhauser comorbidity score (per 1-unit increase)	1.035	(1.033 - 1.037)

Table S2 – b: Association of each factor with development of acute kidney injury stage 1, 2, or 3 vs. no AKI in hospitalized patients

Severity of AKI	Independent variables	OR	(95% CI)
AKI Stage 1	Black race (vs. other)	1.273	(1.211 - 1.337)
	Diabetes (yes vs. no)	1.164	(1.106 - 1.224)
	Obesity (BMI ≥30 vs. <30 kg/m2)	1.104	(1.056 - 1.155)
	Age (per 1-year older)	0.990	(0.989 - 0.992)
	Sex (male vs. female)	1.207	(1.156 - 1.260)
	Baseline eGFR (per 1-unit increase)	0.986	(0.985 - 0.986)
	Elixhauser comorbidity score (per 1-unit increase)	1.031	(1.029 - 1.033)
AKI Stage 2	Black race (vs. other)	1.089	(0.879 - 1.349)
	Diabetes (yes vs. no)	0.789	(0.628 - 0.991)
	Obesity (BMI ≥30 vs. <30 kg/m2)	1.577	(1.310 - 1.898)
	Age (per 1-year older)	0.993	(0.986 - 0.999)
	Sex (male vs. female)	0.854	(0.716 - 1.020)
	Baseline eGFR (per 1-unit increase)	1.004	(1.000 - 1.007)
	Elixhauser comorbidity score (per 1-unit increase)	1.067	(1.060 - 1.074)
AKI Stage 3	Black race (vs. other)	0.995	(0.815 - 1.214)
	Diabetes (yes vs. no)	0.998	(0.811 - 1.227)
	Obesity (BMI ≥30 vs. <30 kg/m2)	1.918	(1.614 - 2.279)
	Age (per 1-year older)	0.994	(0.988 - 1.000)
	Sex (male vs. female)	1.151	(0.975 - 1.359)
	Baseline eGFR (per 1-unit increase)	1.018	(1.014 - 1.021)
	Elixhauser comorbidity score (per 1-unit increase)	1.074	(1.068 - 1.080)

Table S2 – c: Association of each factor with non-recovery of AKI in hospitalized patients who survived to discharge

Independent variables	OR	(95% CI)
Black race (vs. other)	0.847	(0.770 - 0.931)
Diabetes (yes vs. no)	1.021	(0.928 - 1.123)
Obesity (BMI ≥30 vs. <30 kg/m2)	1.274	(1.171 - 1.387)
Age (per 1-year older)	1.011	(1.008 - 1.014)
Sex (male vs. female)	0.759	(0.700 - 0.824)
Baseline eGFR (per 1-unit increase)	1.016	(1.014 - 1.017)
Elixhauser comorbidity score (per 1-unit increase)	1.002	(0.999 - 1.005)

Abbreviations: AKI, acute kidney injury; OR, odds ratio; CI, confidence interval; eGFR, estimated glomerular filtration rate

Models are adjusted for Black race, diabetes, obesity, age, sex, baseline eGFR, Elixhauser comorbidity score, study site

Table S3: Association of Black race, diabetes and obesity with development of AKI stratified by stage and study site in hospitalized patients.

Table S3 – a: The University of Alabama at Birmingham

	**Black vs. non-Black race OR (95%CI)**	**Diabetes vs. no-diabetes OR (95%CI)**	**BMI ≥ vs. < 30 kg/m^2 OR (95%CI)**
N = 27981	9401 (33.6) vs 18580 (66.4)	5731 (20.5) vs 22250 (79.5)	11202 (40.0) vs 16779 (60.4)
**Model 1**			
No AKI	Ref	Ref	Ref
AKI stage 1	1.24 (1.17, 1.31)	1.33 (1.25, 1.42)	1.06 (1.00, 1.12)
AKI stage 2	1.18 (0.91, 1.53)	0.87 (0.62, 1.21)	1.34 (1.05, 1.72)
AKI stage 3	1.24 (0.97, 1.58)	0.87 (0.64, 1.20)	1.15 (0.90, 1.46)
**Model 2**			
No AKI	Ref	Ref	Ref
AKI stage 1	1.34 (1.26, 1.42)	1.11 (1.04, 1.19)	0.95(0.89, 1.01)
AKI stage 2	1.21 (0.93, 1.58)	0.76 (0.54, 1.06)	1.35 (1.04, 1.74)
AKI stage 3	1.05 (0.82, 1.36)	0.96 (0.69, 1.32)	1.25 (0.98, 1.61)
**Model 3**			
No AKI	Ref	Ref	Ref
AKI stage 1	1.27 (1.18, 1.35)	1.12 (1.04, 1.20)	1.03 (0.97, 1.09)
AKI stage 2	1.10 (0.84, 1.43)	0.76 (0.54, 1.07)	1.61 (1.24, 2.10)
AKI stage 3	0.93 (0.71, 1.20)	0.97 (0.70, 1.34)	1.61 (1.25, 2.07)

Table S3 – b: The University of Kentucky Medical Center

	**Black vs. non-Black race OR (95%CI)**	**Diabetes vs. no-diabetes OR (95%CI)**	**BMI ≥ vs. < 30 kg/m^2 OR(95%CI)**
N = 12713			
**Model 1**			
No AKI	Ref	Ref	Ref
AKI stage 1	1.16 (1.02, 1.34)	1.71 (1.56, 1.88)	1.21 (1.10, 1.32)
AKI stage 2	0.89 (0.54, 1.46)	0.89 (0.63, 1.25)	1.15 (0.85, 1.54)
AKI stage 3	0.96 (0.59, 1.54)	1.17 (0.85, 1.61)	1.14 (0.85, 1.53)
**Model 2**			
No AKI	Ref	Ref	Ref
AKI stage 1	1.16 (1.00, 1.33)	1.41 (1.27, 1.55)	1.03 (0.94, 1.13)
AKI stage 2	0.88 (0.53, 1.45)	0.87 (0.61, 1.24)	1.18 (0.87, 1.60)
AKI stage 3	0.93 (0.55, 1.44)	1.26 (0.90, 1.75)	1.26 (0.90, 1.75)
**Model 3**			
No AKI	Ref	Ref	Ref
AKI stage 1	1.15 (1.00, 1.33)	1.41 (1.27, 1.56)	1.17 (1.06, 1.29)
AKI stage 2	0.89 (0.53, 1.47)	0.87 (0.61, 1.25)	1.58 (1.15, 2.16)
AKI stage 3	0.90 (0.55, 1.46)	1.26 (0.90, 1.76)	1.55 (1.14, 2.12)

Table S3 – c: The University of Texas Southwestern

	**Black vs. non-Black race OR (95%CI)**	**Diabetes vs. no-diabetes OR (95%CI)**	**BMI ≥ vs. < 30 kg/m^2 OR(95%CI)**
N = 15362	3103 (20.2) vs 12259 (79.8)	2958 (19.3) vs 12404 (80.7)	5491 (35.7) vs 9871 (64.3)
**Model 1**			
No AKI	Ref	Ref	Ref
AKI stage 1	1.26 (1.14, 1.40)	1.61 (1.46, 1.78)	1.31 (1.20, 1.43)
AKI stage 2	1. 40(0.83, 2.36)	1.15 (0.65, 2.02)	1.20 (0.75, 1.91)
AKI stage 3	1.36 (0.91, 2.05)	1.15 (0.74, 1.78)	2.41 (1.69, 3.44)
**Model 2**			
No AKI	Ref	ref	Ref
AKI stage 1	1.31 (1.17, 1.46)	1.34 (1.20, 1.48)	1.15 (1.05, 1.26)
AKI stage 2	1.24 (0.73, 2.12)	1.20 (0.67, 2.14)	1.13 (0.70, 1.83)
AKI stage 3	0.99 (0.65, 1.51)	1.13 (0.72, 1.77)	2.79 (1.93, 4.02)
**Model 3**			
No AKI	Ref	Ref	Ref
AKI stage 1	1.32 (1.18, 1.47)	1.14 (1.02, 1.27)	1.24 (1.12, 1.36)
AKI stage 2	1.28 (0.75, 2.19)	0.87 (0.48, 1.57)	1.41 (0.86, 2.29)
AKI stage 3	1.05 (0.69, 1.62)	0.79 (0.50, 1.25)	3.78 (2.58, 5.53)

Abbreviations: AKI, acute kidney injury; BMI, body mass index; OR, odds ratio; CI, confidence interval

Model 1 is unadjusted.
Model 2 is adjusted for age, sex, race (when diabetes and obesity were main exposure variables), diabetes (when race and obesity were the main exposure variables), obesity (when race and diabetes were the main exposure variables), and baseline estimated glomerular filtration rate.
Model 3 is adjusted for variables in Model 2 plus the Elixhauser comorbidity score.

Table S4: Association of Black race, diabetes, and obesity with development of AKI in hospitalized patients using pre-admission serum creatinine for baseline definition (sensitivity analysis using the University of Alabama at Birmingham cohort only)

	**Black vs. non-Black race**<br>**OR (95%CI)**	**Diabetes vs. no-diabetes**<br>**OR (95%CI)**	**BMI ≥ vs. < 30 kg/m²**<br>**OR (95%CI)**
N = 13058	4407 (33.7) vs 8651 (66.3%)	3852 (29.5) vs 9206 (70.5)	5106 (39.1) vs 7952 (60.9)
**Model 2**	1.50 (1.38 to 1.63)	1.51 (1.39 to 1.64)	1.19 (1.10 to 1.29)
**Model 3**	1.57 (1.45 to 1.71)	1.55 (1.43 to 1.69)	1.42 (1.31 to 1.54)
**Model 4**	1.64 (1.50 to 1.78)	1.54 (1.41 to 1.67)	1.42 (1.31 to 1.54)

Abbreviations: AKI, acute kidney injury; BMI, body mass index; OR, odds ratio; CI, confidence interval

Model 2 is adjusted for age, sex, study site, Black race (when diabetes and obesity were main exposure variables), diabetes (when race and obesity were the main exposure variables), obesity (when race and diabetes were the main exposure variables), and baseline estimated glomerular filtration rate.
Model 3 is adjusted for all variables in Model 2 plus the Elixhauser comorbidity score.
Model 4 is adjusted for all variables in Model 3 plus the following: pre-admission use of loop diuretics; pre-admission use of angiotensin-converting enzyme inhibitors or angiotensin II receptor blockers; pre-admission use of mineralocorticoid receptor antagonists; the average of mean arterial pressure during the first 48 hours of hospitalization; exposure to nephrotoxins during the first 48 hours of hospitalization; exposure to intravascular contrast agent during the first 48 hours of hospitalization; and admission to a surgical ICU.
Nephrotoxins include aminoglycosides, vancomycin, NSAIDs, calcineurin inhibitors, and amphotericin B.

Table S5: Association of Black race, diabetes, and obesity with development of AKI stratified by stage in hospitalized patients using pre-admission serum creatinine for baseline definition (sensitivity analysis using the University of Alabama at Birmingham cohort only)

	**Black vs. non-Black race OR (95%CI)**	**Diabetes vs. no-diabetes OR (95%CI)**	**BMI ≥ vs. < 30 kg/m^2 OR (95%CI)**
N = 13058	4407 (33.7) vs 8651 (66.3%)	3852 (29.5) vs 9206 (70.5)	5106 (39.1) vs 7952 (60.9)
**Model 2**			
No AKI	Ref	Ref	Ref
AKI stage 1	1.52 (1.39 to 1.67)	1.55 (1.41 to 1.70)	1.18 (1.08 to 1.29)
AKI stage 2	1.58 (1.35 to 1.86)	1.38 (1.17 to 1.63)	1.39 (1.19 to 1.62)
AKI stage 3	1.27 (1.05 to 1.54)	1.46 (1.20 to 1.77)	1.02 (0.85 to 1.23)
**Model 3**			
No AKI	Ref	Ref	Ref
AKI stage 1	1.57 (1.43 to 1.73)	1.58 (1.44 to 1.73)	1.33 (1.22 to 1.46)
AKI stage 2	1.67 (1.42 to 1.97)	1.43 (1.21 to 1.69)	1.75 (1.49 to 2.05)
AKI stage 3	1.40 (1.15 to 1.70)	1.57 (1.29 to 1.92)	1.48 (1.22 to 1.80)
**Model 4**			
No AKI	Ref	Ref	Ref
AKI stage 1	1.60 (1.46 to 1.77)	1.56 (1.41 to 1.71)	1.33 (1.21 to 1.46)
AKI stage 2	1.74 (1.47 to 2.05)	1.38 (1.16 to 1.64)	1.75 (1.49 to 2.05)
AKI stage 3	1.66 (1.36 to 2.03)	1.63 (1.33 to 1.99)	1.54 (1.27 to 1.88)

Abbreviations: AKI, acute kidney injury; BMI, body mass index; OR, odds ratio; CI, confidence interval

Model 2 is adjusted for age, sex, study site, Black race (when diabetes and obesity were main exposure variables), diabetes (when race and obesity were the main exposure variables), obesity (when race and diabetes were the main exposure variables), and baseline estimated glomerular filtration rate.
Model 3 is adjusted for all variables in Model 2 plus the Elixhauser comorbidity score.
Model 4 is adjusted for all variables in Model 3 plus the following: pre-admission use of loop diuretics; pre-admission use of angiotensin-converting enzyme inhibitors or angiotensin II receptor blockers; pre-admission use of mineralocorticoid receptor antagonists; the average of mean arterial pressure during the first 48 hours of hospitalization; exposure to nephrotoxins during the first 48 hours of hospitalization; exposure to intravascular contrast agent during the first 48 hours of hospitalization; and admission to a surgical ICU.
Nephrotoxins include aminoglycosides, vancomycin, NSAIDs, calcineurin inhibitors, and amphotericin B.

Table S6: Association of Black race, diabetes, and obesity with AKI recovery in hospitalized patients who survived to discharge using pre-admission serum creatinine for baseline definition (cohort from the University of Alabama at Birmingham)

Hazard ratios for AKI recovery by multivariate Cox proportional hazards models

	**Black vs. non-Black race** HR (95%CI)	**Diabetes vs. no-diabetes** HR (95%CI)	**BMI ≥ vs. < 30 kg/m^2** HR (95%CI)
N = 3598	1498 (41.6) vs 2100 (58.4%)	1342 (37.3) vs 2256 (62.7)	1547 (43.0) vs 2051 (57.0)
**Model 2**	1.00 (0.92 to 1.09)	0.99 (0.91 to 1.08)	0.96 (0.88 to 1.04)
**Model 3**	0.97 (0.89 to 1.06)	0.97 (0.89 to 1.06)	0.88 (0.81 to 0.96)
**Model 4**	0.97 (0.89 to 1.06)	0.94 (0.86 to 1.02)	0.89 (0.81 to 0.97)

Abbreviations: AKI, acute kidney injury; BMI, body mass index; OR, odds ratio; HR, hazard ratio; CI, confidence interval

Model 2 is adjusted for age, sex, study site, Black race (when diabetes and obesity were main exposure variables), diabetes (when race and obesity were the main exposure variables), obesity (when race and diabetes were the main exposure variables), and baseline estimated glomerular filtration rate.
Model 3 is adjusted for all variables in Model 2 plus the Elixhauser comorbidity score.
Model 4 is adjusted for all variables in Model 3 plus the following: exposure to loop diuretics prior to the onset of AKI during hospitalization; exposure to angiotensin-converting enzyme inhibitors or angiotensin II receptor blockers prior to the onset of AKI during hospitalization; exposure to mineralocorticoid receptor antagonists prior to the onset of AKI during hospitalization; the average of mean arterial pressure during the first 48 hours of hospitalization; exposure to nephrotoxins during the first 48 hours of hospitalization; exposure to intravascular contrast agents during the first 48 hours of hospitalization; admission to a surgical ICU; and length of hospital stay.
Nephrotoxins include aminoglycosides, vancomycin, NSAIDs, calcineurin inhibitors, and amphotericin B.

STROBE Statement - Checklist

	Item No	Recommendation	Page No
**Title and abstract**	1	(*a*) Indicate the study's design with a commonly used term in the title or the abstract	1-2
		(*b*) Provide in the abstract an informative and balanced summary of what was done and what was found	2
**Introduction**			
Background/rationale	2	Explain the scientific background and rationale for the investigation being reported	3
Objectives	3	State specific objectives, including any prespecified hypotheses	3
**Methods**			
Study design	4	Present key elements of study design early in the paper	3
Setting	5	Describe the setting, locations, and relevant dates, including periods of recruitment, exposure, follow-up, and data collection	3-4
Participants	6	(*a*) Give the eligibility criteria, and the sources and methods of selection of participants. Describe methods of follow-up	3-4
		(*b*) For matched studies, give matching criteria and number of exposed and unexposed	-
Variables	7	Clearly define all outcomes, exposures, predictors, potential confounders, and effect modifiers. Give diagnostic criteria, if applicable	4-6
Data sources/ measurement	8*	For each variable of interest, give sources of data and details of methods of assessment (measurement). Describe comparability of assessment methods if there is more than one group	4-6
Bias	9	Describe any efforts to address potential sources of bias	4-7
Study size	10	Explain how the study size was arrived at	
Quantitative variables	11	Explain how quantitative variables were handled in the analyses. If applicable, describe which groupings were chosen and why	4-6
Statistical methods	12	(*a*) Describe all statistical methods, including those used to control for confounding	6-7
		(*b*) Describe any methods used to examine subgroups and interactions	6-7
		(*c*) Explain how missing data were addressed	-
		(*d*) If applicable, explain how loss to follow-up was addressed	-
		(*e*) Describe any sensitivity analyses	-
**Results**			
Participants	13*	(a) Report numbers of individuals at each stage of study—eg numbers potentially eligible, examined for eligibility, confirmed eligible, included in the study, completing follow-up, and analysed	8<br>Fig. 1
		(b) Give reasons for non-participation at each stage	Fig. 1
		(c) Consider use of a flow diagram	Fig. 1
Descriptive data	14*	(a) Give characteristics of study participants (eg demographic, clinical, social) and information on exposures and potential confounders	8<br>Table 1
		(b) Indicate number of participants with missing data for each variable of interest	-
		(c) Summarise follow-up time (eg, average and total amount)	-
Outcome data	15*	Report numbers of outcome events or summary measures over time	8<br>Fig. 2

Main results	16	(*a*) Give unadjusted estimates and, if applicable, confounder-adjusted estimates and their precision (eg, 95% confidence interval). Make clear which confounders were adjusted for and why they were included	9-10<br>Fig. 3-5
		(*b*) Report category boundaries when continuous variables were categorized	5-6
		(*c*) If relevant, consider translating estimates of relative risk into absolute risk for a meaningful time period	-
Other analyses	17	Report other analyses done—eg analyses of subgroups and interactions, and sensitivity analyses	Table S2<br>Table S3
**Discussion**			
Key results	18	Summarise key results with reference to study objectives	10-11
Limitations	19	Discuss limitations of the study, taking into account sources of potential bias or imprecision. Discuss both direction and magnitude of any potential bias	12
Interpretation	20	Give a cautious overall interpretation of results considering objectives, limitations, multiplicity of analyses, results from similar studies, and other relevant evidence	11-13
Generalisability	21	Discuss the generalisability (external validity) of the study results	12-13
**Other information**			
Funding	22	Give the source of funding and the role of the funders for the present study and, if applicable, for the original study on which the present article is based	14
